# Computed tomography for managing periprosthetic femoral fractures. A retrospective analysis

**DOI:** 10.1186/s12891-019-2632-y

**Published:** 2019-05-29

**Authors:** Markus Rupp, Stefanie Kern, Abdullah Ismat, Thaqif El Khassawna, Gero Knapp, Gabor Szalay, Christian Heiss, Christoph Biehl

**Affiliations:** 1 Department of Trauma, Hand and Reconstructive Surgery, University Hospital of Giessen-Marburg, Giessen, Germany; 2 Experimental Trauma Surgery, Faculty of Medicine, Justus-Liebig University of Giessen, Giessen, Germany

**Keywords:** Computed tomography, Periprosthetic fracture, Femur, Arthroplasty

## Abstract

**Background:**

Periprosthetic fractures (PPF) present a common cause for revision surgery after arthroplasty. The choice of performing either an osteosynthesis or revision arthroplasty depends on the orthopedic implant anchored and loosening. Standard diagnostics include x-ray imaging. CT is usually performed to confirm implant loosening in case of ambiguous diagnosis on standard x-ray imaging. This study aimed to examine the role of CT as a diagnostic modality and its implications for treatment planning and outcome.

**Methods:**

Patients treated for PPF from January 2010 to February 2018 were included. X-ray and CT reports were analyzed to assess implant loosening. The planning for surgery and the final surgical treatment were evaluated. In addition, patient characteristics were analyzed and compared between patients with and without additional CT as a preoperative diagnostic procedure.

**Results:**

Seventy-five patients were eligible for the study. X-ray imaging was performed in 90.7% of cases. CT was performed in 60% of the cases as part of the preoperative diagnostic. A clear statement on implant stability or loosening could not be made in 69.1% after X-ray imaging and in 84.4% following CT imaging. Revision arthroplasty for loosened femoral prosthesis components was necessary in 40% of cases. No difference could be determined comparing patients with X-ray imaging to those with X-ray and additional CT. In both groups, operative treatment did not deviate from the preoperative planning.

**Discussion:**

In two thirds of the conventional radiographic findings, no reliable evaluation of implant loosening was possible in femoral PPFs. Intriguingly, additional CT did not improve the evaluation of implant loosening. Nonetheless, CT scans are often performed if loosening assessment is unclear on regular radiographs. This fact can explain the bias CT results in comparison to regular radiography. However, software-supported CT diagnosis could help to adequately answer the question of loosened implants in PPF in the near future. Since the diagnosis of fracture and their morphology assessment is currently adequately performed using X-rays, CT shall not be considered as the gold standard.

## Background

One of the biggest complications after complete joint replacement are periprosthetic fractures (PPFs). After complete knee replacement, PPFs are the fourth most common cause of revision surgery. Only aseptic and septic loosening as well as instabilities lead to more revisions [1, 2]. Data from the Swedish National Hip Replacement Registry show that PPFs are the second most common reason for revision of arthroplasty 4 years after primary hip replacement [3]. For short shaft hip endoprosthesis, PPFs were given as the main reason (50%) for reoperation in the first 2 years after the hip endoprosthesis [4]. A further increase in periprosthetic fractures is expected as demographic ageing in the Western World is accompanied by an increase in joint replacement surgery [5]. PPF presents a challenge for both patients and surgeons. On the one hand, morbidity and mortality in patients with PPFs are high. On the other hand, surgical treatment needs advanced skills not only in revision arthroplasty, but also in trauma surgery. Revision surgery is generally associated with higher complication rates. Therefore, the clear definition of operative strategy in advance is needed. Depending on the classification of the PPFs, treatment algorithms for osteosynthesis or revision arthroplasty are recommended. In 2014, Duncan and Haddad published the Unified Classification System (UCS), a comprehensive PPF classification system [6] that included earlier classification systems such as the Vancouver Classification [7]. Based on the UCS, treatment recommendations are given. However, there is no evidence-based algorithm for PPF diagnosis. Conventionally, x-rays are the first-line imaging tests. A CT scan is recommended as a further diagnostic modality to assess fracture morphology, implant stability and bone stock [8]. The CT provides detailed images of cortical and trabecular bone. In addition, 3D reconstruction provides an easy-to-understand visualization of fracture morphology. Added costs, radiation exposure and stress associated with the additional needed examination for the patient with a femur fracture are accepted on the assumption that CT helps to decide the surgical strategy. In implant loosening cases, arthroplasty is needed, while osteosynthesis should be performed in fixed prosthetic implants [6]. The aim of this study was to explore the role of CT in preoperative planning and impact on treatment result.

## Methods

All included patients suffered from periprosthetic femur fractures after either hip or knee arthroplasty. The study was a single center study. Patients had to be treated between January 2010 and February 2018. The local ethical committee of the medical faculty approved the data used in this study.

We performed a retrospective analysis of all postoperative femoral PPFs. All intraoperative fractures during arthroplasty were excluded from the analysis. Further exclusion criteria were all other periprosthetic fractures such as acetabular fractures in hip prostheses and tibial fractures in knee prostheses. Patients who underwent revision because of mechanical complications, periprosthetic joint infection and aseptic loosening were also excluded. The authors examined whether X-ray or CT scans were performed as preoperative diagnostics. Each patient with an X-ray received a pelvic overview image and a lateral image of the affected side. All CT scans were performed without contrast agents. The reports from X-rays and CT scans were evaluated to ascertain the detection of implant loosening. For that purpose, radiological reports were analyzed by the authors. The reports evaluation was graded as “loose” or “fixed”. In addition, we analyzed the surgical planning and fracture classification, which was approved by an experienced orthopedic trauma surgeon. Last, the final surgical procedures and the intraoperative assessment of prosthetic loosening were evaluated. We divided the procedures performed into either osteosynthesis for stable prosthetic implants or revision arthroplasty for loose prosthetic implants. Osteosynthesis procedure included plating, cabling and intramedullary nailing with a custom nail was previously established in our clinic for stable implant [9]. We analyzed patient records based on demographic and treatment-relevant data: 1) gender; 2) age at time of PPF; 3) weight; 4) height; 5) body mass index; 6) American Society of Anesthesiologists (ASA) score; 7) number of secondary diagnoses; 8) length of hospital stays; 9) time to surgery after trauma; and 10) early fixation method of the implant (cement-less or cemented).

The data were analysed with SPSS Statistics Version 25.0 (IBM, SPSS Inc., Armonk, NY). Collected data sets from clinical data were examined with descriptive statistics for their normality. The significance analysis for the nonparametric distribution was performed with the Mann-Whitney U-Test or the Wilcoxon signed rank sum test. Frequencies were calculated. For the analysis of differences between patients with and without CT, the Chi-square test (χ^2^)- or Fisher’s more accurate test for categorical variables was applied. The Mann-Whitney U-test was used for comparisons between the groups. The threshold for significance was set to *p* < 0.05. The data were plotted as mean ± standard error of the mean (SEM).

## Results

**Fig. 1:**
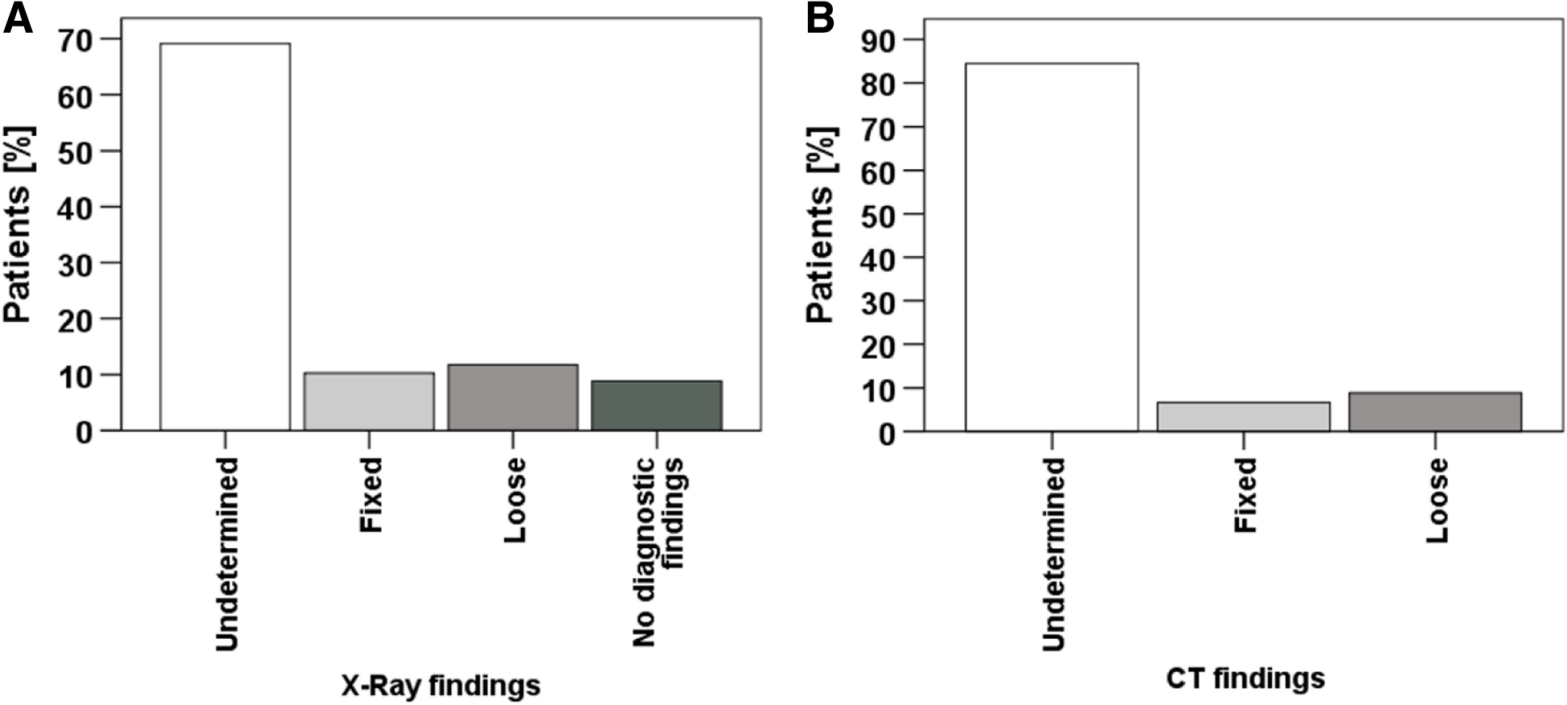
**a** Analysis of X-ray findings for loosening of the implant in femoral PPFs. In the majority of the patients a reliable analysis of implant loosening was not possible (**b**) Analysis of CT scan findings for loosening of the implant in femoral PPFs. In more than 80% of the cases implant loosening could not be determined

**Fig. 2:**
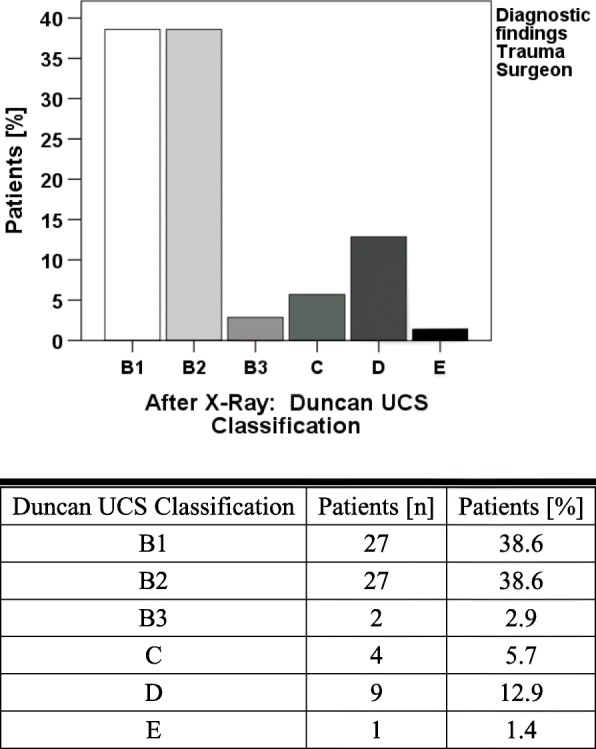
Femoral periprosthetic fractures were most often diagnosed as B1 and B2 fractures according to the Unified Classification System prior to surgery. Classification was based on both X-ray and CT images and performed by experienced trauma surgeons

In the given time frame 1015 patients were treated with the proper ICD codes. After reviewing all patient files, 75 patients treated with PPF were included in the evaluation. Women were affected in 2/3 of the cases (50 of 75 cases). The average age at injury was 79.2 years (46–98 years), the average lifetime of the prosthesis 16.5 years (0–36.5 years). 60.8% of all prostheses were cemented, 5.3% of hip prostheses were hemi prostheses and 4.0% were revision prostheses. X-ray imaging as basic diagnosis was performed in 90.7% of all cases. In 9.3% of cases, a CT scan was already performed during emergency diagnosis (Table 1). Altogether 60% of the patients in the examined group underwent a CT scan. In 69.1% of cases, X-ray findings alone failed to provide a clear suggestion of loosened prosthesis. CT scans evaluation showed that 84.4% of cases failed to give a clear statement about the prosthesis loosening (Fig. 1, Table 2). Before surgery, the fractures were classified according to UCS by experienced trauma surgeons. Revision arthroplasty was performed in 40% of all PPFs. Preserving the prosthesis through various osteosynthesis procedures was possible in 60% of the cases. Most fractures were classified as B1 and B2 with 38.6% each. Type C fractures occurred in 5.7%, D fracture in 12.6% and E fracture in 1.4% (Fig. 2). An operative strategy was chosen based on the classification. In 2.7% of the cases reduction and retention were performed with cerclages, 5.3% were treated by fixation with a customized intramedullary nail [9] and 52% by plate fixation.

**Table 1:** Demographic data of the included patient cohort

		CT [n / %]	No CT [n / %]	Pearsonsχ^2^ [Value / df]	*p*-value
Total		45 / 60.0	30 / 40.0		
Gender	Female	28 / 62.2	22 / 73.3	1.000 / 1	0.454
	Male	17 / 37.8	8 / 26.7		
Cement	No	19 / 42.2	10 / 34.5	0.443 / 1	0.627
	Yes	26 / 57.8	19 / 65.5		
Prosthesis	Hemi-Prosthesis	2 / 4.4	2 / 6.7	0.919 / 2	1.000
	Full- Prosthesis	41 / 91.1	27 / 90.0		
	Revision-Prosthesis	2 / 4.4	1 / 3.3		
ASA	1	1 / 2.9	–	4.827 / 3	0.141
	2	12 / 34.3	11 / 45.8		
	3	22 / 62.9	11 / 45.8		
	4	–	2 / 8.3		
Operative Strategy	Cerclage	–	2 / 6.7	6.838 / 3	0.056
	Plate Fixation	21 / 53.8	18 / 60.0		
	Exchange	20 / 44.4	10 / 33.3		
	Intramedullary Nail	4 / 8.9	–		
Intraoperative	Fixed	24 / 54.5	17 / 58.6	0.118 / 1	0.812
	Loose	20 / 45.5	12 / 41.4		

Numbers (n) and percentage (%) are presented in the right column

**Table 2:** Overview of diagnostic findings of X-ray, CT and the combination of both. In addition, intraoperative findings are presented.

		[n / %]
X-Ray	Undetermined	47 / 69.1
	Fixed	7 / 10.2
	Loose	8 / 11.8
	No diagnostic findings	6 / 8.8
CT	Undetermined	38 / 84.4
	Fixed	3 / 6.7
	Loose	4 / 8.9
X-Ray & CT	Undetermined	28 / 73.7
	Fixed	2 / 5.3
	Loose	2 / 5.3
	Difference between X-Ray & CT	6 / 15.8
Preoperative Diagnosis	Fixed	46 /61.3
	Loose	29 / 38.7
Intraoperative Diagnosis	Fixed	41 / 54.7
	Loose	32 / 42.7

In addition, intraoperative findings are presented

There was no difference in the number of CT scans performed for sex (χ^2^ (1) =1.000, *p* = 0.454), cemented or non-cemented prosthesis (χ^2^ (1) =0.443, *p* = 0.627) or a particular type of prosthesis (χ^2^(2) =0.949, *p* = 1.000). In addition, no significant difference in surgical treatment was noted. No changes in surgical strategy were documented in the operation reports (χ^2^(3) =6.838, *p* = 0.056). The average hospital stay was 21 days (+/− 10 days). The length of the patient stay was not influenced by an additional CT scan. Patients who had undergone a preoperative CT did not wait any longer for the operation. Reasons for delayed surgery were primarily morbidity and secondary diagnoses. Patients had an average of 3.5 secondary diagnoses and an average ASA score of 2.6. There was no difference in ASA score between patients with and without CT (χ^2^ (3) =4.827, *p* = 0.141). Cardiac examination was necessary in 10 patients before surgery.

## Discussion

Our analysis revealed that reports of X-rays and CT scans often do not provide enough information about possible implant loosening in femoral PPF. In 60% of the X-rays and 80% of the CT scans, no reliable statement could be made about implant loosening. Since implant stability is difficult to assess for both X-ray and CT scans, our results are in line with findings with reported inter- and intra-observer reliability and validity of the Vancouver classification system. For this classification system, which is well-known and widespread within the orthopedic community, low levels of reliability have been reported. Naqvi and coworkers report Kappa values of 0.61–0.69 for the interobserver agreement and 0.74–0.90 for the intra-observer agreement for the entire Vancouver classification system [10]. Baba and colleagues could explain poorer interobserver reliability in Vancouver classification-based assessment with 0.41 for X-rays and 0.48 for CT scans [11]. Cohen’s kappa value of 0.6–0.79 represents a moderate agreement (35–63% of the data are reliable), while a kappa value of 0.40–0.59 represents a weak agreement, meaning that only 15–35% of the data are reliable [12]. About implant loosening, the higher rates of ambiguous CT scans may be because of distortion, as more CT scans are performed in patients where loosening was difficult to assess in X-ray diagnostics. The need of carrying out CT scans must be justified as indispensable for the decision on therapy. The repositioning for examining patients with dislocated femur fractures carries the risk of nerve and vascular injuries caused by dislocated fracture fragments. In addition, the additional examination can once again place a heavy load on patients who are already suffering from noticeable pain and discomfort. This should be considered especially in treating fragile, elderly patients.

For radiation exposure, the dose in older patients, who represent the majority in PPF, is considered insignificant for the carcinogenic effect. However, to reduce the cancer risk associated with medical imaging irradiation, a stricter suggestion is needed, especially for younger patients [13]. The reasons for CT may be better fracture morphology assessment and estimation of bone quality. However, assessment of bone quality by CT seems to be difficult. The prevalence of B2 and B3 fractures in our study (38.6% vs. 2.7%) was different in comparison to the reported numbers in other studies with similar patient collectives (B2 17.2% vs. B3 13.2%) [14, 37% vs. 5%) [15]. This leads to the assumption that the difficulty in distinguishing between good and poor bone quality on imaging may be reason for the observed difference of reported numbers of B2 and B3 fractures. In this case, quantitative CT-based measurements would be helpful. Metal artifacts reduce the diagnostic value of CT’s [8]. Such artifacts can result in damage of the informative value while assessing implant stability. Special software can help to better evaluate bone quality and reduce artifacts around the implants.

The demographic figures of our collective by age and sex of patients are comparable to those in the literature [14, 16, 17]. The length of hospital stay was comparable to a cohort of PPF patients in Ireland [17]. An average time of 6 days to surgery after admission was somewhat later, as Sellan et al. reported with a duration of 4 days. The data showed no influence of time of fixation or revision of the arthroplasty on the duration of the hospital stay and the one-year mortality. Since the additional CT has no influence on length of hospital stay, the additional diagnostics does not bring any additional benefit for management of PPF.

The intraoperative examination of the implant loosening is based on a trivial mechanical examination. If the prosthesis needs to be exposed intraoperatively due to fracture morphology, stability can be easily checked. However, if the implant is not easily accessible or exposure is not primarily necessary, no reliable statement can be made intraoperatively about possible implant loosening. Finally, this may lead to different intraoperative findings compared to the implant stability assessed preoperatively by imaging. Besides manual mechanical testing, a practical tool is needed for the intraoperative examination of prosthesis loosening, especially in uncertain radiological findings. Resonance frequency analysis, an established method that tests the loosening of dental implants and is easy to perform, could contribute to better assessment intraoperatively [18]. While revision arthroplasty of fixed prostheses may lead to an unnecessarily more complex operation, erroneously performed osteosynthesis in implant loosening may lead to complications and necessary follow-up procedures.

The limitation of the present study is its retrospective design. The study cohort is therefore inhomogeneous. All different kinds of femoral orthopedic implants in patients suffering from PPF have been included to the study. Also, retrospective design not only results in changes in CT protocols over the study period of 8 years but also in differences in X-ray machines, CT scanners, emitted radiation and associated picture quality. Different radiologists as well as trauma and orthopedic surgeons diagnosing X-rays, CTs, the latter planning treatment on those findings, have to be mentioned as well. This study presents an unavoidable problem of no output value of implant loosening. Nonetheless, this clinical diagnostic study resembles daily routine and problems both surgeons as well as radiologists are faced with each day. A prospective study using standardized approach would be necessary to consolidate the results of this study to overcome the drawbacks.

## Conclusion

CT does not improve preoperative diagnostics for implant stability in femoral PPF. Planning the strategy operative treatment, choice of treatment, and the duration of hospitalization were not correlative to additional CT. Nonetheless, Software-supported CT diagnostics can help to sufficiently answer the question of loosened implants and quantify bone quality in future. The authors recommend orthopedic surgeons to rely on native radiographs for fracture diagnostics. CT diagnostics should only be considered, if assessment of fracture morphology is not possible on high quality radiographs. Avoiding the additional CT scans will also help to reduce the involved costs, radiation exposure and additional associated stress for the patients.

## Abbreviations

ASA: American Society of Anesthesiologists
CT: Computed tomography
ICD: International classification of diseases
PPF: Periprosthetic fracture
SPECT: Single-photon emission computed tomography
UCS: Unified classification system
